# Sleep Disturbances in Generalized Anxiety Disorder: The Role of Calcium Homeostasis Imbalance

**DOI:** 10.3390/ijerph20054431

**Published:** 2023-03-01

**Authors:** Elvira Anna Carbone, Giulia Menculini, Renato de Filippis, Martina D’Angelo, Pasquale De Fazio, Alfonso Tortorella, Luca Steardo

**Affiliations:** 1 Department of Medical and Surgical Sciences, University Magna Graecia, 88100 Catanzaro, Italy; 2 Department of Psychiatry, University of Perugia, Piazzale Lucio Severi, 1, 06132 Perugia, Italy; 3 Department of Health Sciences, University “Magna Graecia”, 88100 Catanzaro, Italy

**Keywords:** anxiety disorder, calcium, parathyroid hormone, vitamin D, sleep, insomnia

## Abstract

Patients with a generalized anxiety disorder (GAD) often report preeminent sleep disturbances. Recently, calcium homeostasis gained interest because of its role in the regulation of sleep–wake rhythms and anxiety symptoms. This cross-sectional study aimed at investigating the association between calcium homeostasis imbalance, anxiety, and quality of sleep in patients with GAD. A total of 211 patients were assessed using the Hamilton Rating Scale for Anxiety (HAM-A), Pittsburgh Sleep Quality Index questionnaire (PSQI) and Insomnia Severity Index (ISI) scales. Calcium, vitamin D, and parathyroid hormone (PTH) levels were evaluated in blood samples. A correlation and linear regression analysis were run to evaluate the association of HAM-A, PSQI, and ISI scores with peripheral markers of calcium homeostasis imbalance. Significant correlations emerged between HAM-A, PSQI, ISI, PTH, and vitamin D. The regression models showed that patients with GAD displaying low levels of vitamin D and higher levels of PTH exhibit a poor subjective quality of sleep and higher levels of anxiety, underpinning higher psychopathological burden. A strong relationship between peripheral biomarkers of calcium homeostasis imbalance, insomnia, poor sleep quality, and anxiety symptomatology was underlined. Future studies could shed light on the causal and temporal relationship between calcium metabolism imbalance, anxiety, and sleep.

## 1. Introduction

Sleep is a basic human need and is essential for good health, well-being, and good quality of life. We spend nearly a third of our life sleeping. However, people often experience difficulties in sleeping that may become disabling and result in daytime dysfunction [1,2,3]. According to the third edition of the International Classification of Sleep Disorders (ICSD-3), insomnia is characterized by difficulty in either initiating, maintaining, or continuing sleep, despite the adequate opportunity and condition for sleep. Nowadays, insomnia represents the most common sleep disorder [4,5] affecting especially women and older people, and it coexists very frequently with general health problems (e.g., cardiovascular diseases, chronic pain syndrome, diabetes, obesity, asthma) [6]. Sleep disturbances are commonly detected in the general population and individuals with psychiatric disorders [7]. Considering that sleep can affect mental health, having a psychiatric disorder, in turn, could impact on sleep quality. Studies indicate that insomnia very often coexists with psychiatric disorders [8]. Particularly, insomnia is most frequently associated with major depression or an anxiety disorder, mainly, generalized anxiety disorder (GAD) [9].

About 60–70% of patients with GAD and panic disorder reported prominent sleep disturbances [9], leading to a negative impact on functioning and quality of life [10] and the course and treatment of psychiatric illness [11].

Sleep–wake regulation is classically described as resulting from the interaction of circadian and homeostatic processes [12], which in turn influence the opposite activity of neurons stimulating wakefulness and neurons stimulating sleep [13]. The dysregulation of this process and consequent insomnia seems to be linked to the alteration of different hormones such as insulin, cortisol, leptin, orexin, ghrelin or growth factor, and vitamin D [14,15,16,17,18,19,20].

In recent years, calcium homeostasis has received increasing interest, with research supporting the role of parathyroid hormone (PTH), vitamin D (Vit D), and calcium (Ca^++^) in mental health conditions [21]. Vit D, together with PTH, regulates the homeostasis of Ca^++^, modulating calcium transportation in the gut, bone, and kidney and the immune modulation, the antioxidant defense system, and several inflammatory processes [22,23,24]. By appropriate actions of Vit D and PTH, Ca^++^ is maintained in the range or promptly corrected if necessary. An alteration or defect of any of this system results in the calcium homeostasis imbalance. It was already demonstrated in schizophrenia [25], depression [26], bipolar disorder [27], anxiety [28,29,30], and sleep disorders [31,32,33,34,35].

This could be explained considering different activities of Vit D, Ca^++^, and PTH. Vit D receptors are widely expressed in all human bodies and brain [36,37,38,39] and their increased expression was demonstrated in specific brain regions involved in anxiety and sleep regulation, such as the prefrontal cortex and the limbic system [40,41]. In these areas, particularly in the prefrontal cortex [42], Vit D can directly increase the biosynthesis of dopamine/noradrenaline and serotonin [43,44,45,46], and improve the expression of the growth factor hormone and the BDNF [47,48,49]. Ca^++^ is very important in the central nervous system (CNS) as a cofactor, second messenger, and signaling molecule, and for transmitters release [50]. Additionally, PTH contributes to neuronal homeostasis [51] regulating circulating and intracellular calcium levels in the CNS [52].

Vit D has gained prominence due to its antioxidant, anti-inflammatory, pro-neurogenic, and neuromodulator properties that appear to be fundamental to its anxiolytic properties [53,54,55,56]. Data are supported by studies demonstrating that supplementation of Vit D can improve anxiety symptoms, [57,58,59] as well as sleep disorders and sleep quality [60]. On the other hand, experimental evidence has shown that Ca^++^ signaling plays a crucial role in regulating sleep–wake rhythms [61]. There is also evidence suggesting that increased dietary Ca^++^ intake improves anxiety [62], quality of sleep, and reduces insomnia [63,64]. Interestingly, total Ca^++^ presents a diurnal variability during normal sleep [65], underlining the role in regulating sleep duration in mammals [66], possibly due to the involvement in producing melatonin from tryptophan in the brain [67].

Although several studies investigated the co-occurrence of sleep disturbances and anxiety disorders [68,69], showing that the relationship between these two conditions is particularly complex [70], few studies focused on calcium homeostasis imbalance and data are not conclusive. Therefore, such experimental evidence led clinicians to comprehensively investigate the effect of calcium metabolism imbalance on anxiety disorders.

Based on the above, the current study aimed at investigating the association between calcium imbalance through the determination of Ca^++^, Vit D, and PTH levels, anxiety psychopathology severity, and altered hypnic pattern in a sample of patients suffering from a generalized anxiety disorder. Thus, the current study tries to explore whether calcium metabolism imbalance could be associated with sleep quality and worsening of symptoms in patients with anxiety disorders. Therefore, the aims of the present study are (1) to identify the association between calcium imbalance and quality of sleep in patients suffering from generalized anxiety disorder (GAD) and (2) to evaluate how this association may impact illness severity in patients suffering from GAD.

## 2. Materials and Methods

Consecutive outpatients were screened for eligibility at the Psychiatric Unit of the University Hospital Mater Domini in Catanzaro from May 2020 to July 2022. Inclusion criteria were age between 18 and 75 years; primary diagnosis of GAD according to the Diagnostic and Statistical Manual of Mental Disorders, Fifth Edition (DSM-5) [8]; and willingness to participate in the study. Participants were considered not eligible in cases of an inability to provide a written informed consent to participate in the study; presence of moderate or severe cognitive impairment as assessed at the first contact visit by Mini-Mental State Evaluation (MMSE) ≤22 [71]; comorbidity with neurologic diseases, endocrinological diseases (hypo/hyperparathyroidism), or substance and/or alcohol use disorders; pregnancy or post-partum period; current treatment with medications that can alter calcium metabolism, such as Vit D supplementation or calcium phosphonate or bisphosphonates.

Patients presenting comorbid depressive features were not excluded, considering the high prevalence of anxiety and depressive symptoms co-occurrence in clinical practice. However, we excluded patients with a severe or subthreshold depressive condition clinically evaluated at the moment of the enrollment.

All participants meeting the inclusion/exclusion criteria were recruited and included in the study after receiving a full description of the study aims and design and obtaining their written informed consent to participate in the study. The Structured Interview for DSM-5 Disorders, Clinician Version (SCID-5-CV) [72] was used for the diagnosis. All tests were performed by experienced psychiatrists who were trained in the administration of neuropsychiatric tests and used these tools in their daily clinical practice.

The study was carried out following the latest version of the Declaration of Helsinki and the protocol approval was obtained by the Ethics Committee of the University of Catanzaro (307/2020).

### 2.1. Procedures and Measures

Patients’ socio-demographic and clinical characteristics were collected using an ad hoc schedule evaluating sex, age, civil status, education, employment status, family history of psychiatric illnesses, and age at onset of the disorder.

#### 2.1.1. Psychological Measures

Participant answered the following scales:

- Hamilton Rating Scale for Anxiety (HAM-A) [73], to assess the clinical severity of anxiety symptoms. The scale consists of 14 items scoring on a scale of 0 (not present) to 4 (severe). Each item is defined by a series of symptoms, and measures both psychic anxiety (mental agitation and psychological distress) and somatic anxiety (physical complaints related to anxiety). The total score ranges from 0 to 56, where <17 indicates mild severity, 18–24 mild to moderate severity, and 25–30 moderate to severe. Cronbach’s alpha was 0.934 in this study.
- Pittsburgh Sleep Quality Index Questionnaire (PSQI), to analyze sleep quality. The self-reported questionnaire is made up of 19 items, used to create seven components with a score ranging between 0 (no problem) and 3 (major problem), namely, subjective sleep quality (hereafter referred to as Quality), sleep latency (Latency), sleep duration (Duration), habitual sleep efficiency (Efficiency), sleep disturbances (Disturbances), use of sleeping medication (Medication), and daytime dysfunction (Dysfunction). The total score from these seven components varies between 0 (no problem) and 21 (major problem). A global score of ≥5 is used to identify people with poor sleep quality [74,75]. People with a score of 5 or higher, experienced poor sleep quality, and those with a score of less than 5 experienced good sleep quality. Cronbach’s alpha was 0.77 [76]. Cronbach’s alpha was 0.834 in this study.
- Insomnia Severity Index (ISI), to assess the nature, severity, and impact of sleep difficulties in the last 2 weeks. A 5-point Likert scale is used to rate the 7 items, with scores ranging 0–28 that yield four categories: absence of insomnia (0–7); subthreshold insomnia (8–14); moderate insomnia (15–21); and severe insomnia (22–28) [77]. Cronbach’s alpha was 0.784 in this study.

#### 2.1.2. Biological Measures

Serum levels of calcium (mmol/L), 25-OH-vitamin D (ng/mL), and PTH (pg/mL) were assessed in the same laboratory to ensure standardized procedures. Blood samples were collected from all patients at recruitment after 12–14 h fasting.

Calcium was measured using standard laboratory methods. Blood was centrifuged, and serum was stocked at −30 °C for α,25 (OH)_2_ vitamin D and PTH and evaluated by chemiluminescence immunoassays using adequate kits (Diasorin Liaison; ADVIA Centaur). According to the Endocrine Society’s Clinical Practice Guideline, Vit D deficiency was considered when its values were <20 ng/mL; insufficiency between 21–29 ng/mL; and sufficiency between 30–100 ng/mL [78]. Levels of Ca^++^ between 8.9 and 10.01 mg/dL are considered normal, whilst the range 15–55 pg/mL is considered normal for the PTH.

Levels of Vit D < 20 ng/mL, Ca^++^ < 8.8 mg/dL or >10 mg/dL, and PTH < 15 pg/mL or >55 pg/mL were the cut-off considered for calcium homeostasis imbalance (Table 1).

### 2.2. Statistical Analysis

Descriptive statistics were calculated for socio-demographic and clinical characteristics, as well as for scores at relevant assessment instruments. The quantitative variables were expressed as mean and standard deviation (SD) and the qualitative variables as frequency and percentage (%).

A Spearman correlation analysis was used to assess the relationship between sleep quality, anxiety symptoms, and calcium homeostasis imbalance. Linear regression analysis was performed to further investigate the relationship between sleep quality, anxiety, and calcium homeostasis imbalance using PSQI, ISI, and HAM-A scores as dependents variables and PTH, calcium, and Vit D as independent variables. All tolerance values in the regression analyses were >0.1 and all variance inflation factors were <10, expressing that the assumption of multicollinearity was not violated. The *p*-value < 0.05 was considered significant in this study. Data were analyzed with the Statistical Package for Social Sciences Version 26 (SPSS, Chicago, IL, USA) [79].

## 3. Results

Overall, 211 participants suffering from GAD met the inclusion/exclusion criteria and were enrolled in the study. The average age (±standard deviation, SD) was 46.9 (±13.8). Most of the participants were female (51%), married (45.5%), graduated (76%), employed (63%), and with positive family history for psychiatric disorders (64.5%). The mean age at onset was 27.8 ± 11.1. The mean of HAM-A total, PSQI total, ISI total was 25.6 ± 13.7, 10.96 ± 6.2 and 14.36 ± 8.2, respectively. Indices of calcium metabolism showed a normal calcium level 9.5 ± 0.4, higher PTH level (54.6 ± 20.5), and lower Vit D level (29.4 ± 25.1) (Table 2).

Table 3 includes the results of Spearman’s correlations between HAM total score, PSQI subscales and total score, ISI total score, calcium, PTH, and Vit D. Significant correlations emerged for all the variables, with the sole exception of Ca^++^.

A linear regression analysis was performed to assess the association between calcium imbalance, anxiety symptoms, and quality of sleep. In the three models, PSQI total, HAM-A total, and ISI total, respectively, were selected as dependent variables and PTH, Vit D, and Ca^++^ as independent variables. In the first model, higher PTH levels and lower Vit D levels (R^2^ = 0.603; F = 80.752; *p* < 0.001) predicted PSQI total; in the second model, higher PTH levels and lower Vit D levels predicted HAM-A total (R^2^ = 0.685; F = 115.137; *p* < 0.001), and in the last model, higher PTH levels and lower Vit D levels predicted ISI total (R^2^ = 0.672; F = 105.516; *p* < 0.001). Thus, an imbalance of PTH and Vit D levels predicted insomnia, higher levels of anxiety, and poor quality of sleep. See Table 4.

## 4. Discussion

This study found a strong relationship between calcium homeostasis imbalance, poor sleep quality, and anxiety symptomatology in patients suffering from GAD. To the best of our knowledge, this is the first study aimed at investigating the association between calcium homeostasis imbalance and quality of sleep in patients with GAD. The study findings suggest that patients with GAD and low levels of Vit D and higher levels of PTH exhibit insomnia, poor quality of sleep, and higher levels of anxiety, highlighting its impact on the psychopathological burden.

A growing body of literature focused on the calcium imbalance in psychiatric disorders [21,25,27,28,29,30,31,32,33,34,35] and our results are in line with them. In our sample, significant correlations emerged for PSQI, HAM-A, ISI, PTH, and Vit D. The association between poor sleep quality and high levels of PTH and low levels of Vit D may be read considering the sleep–wake dysregulation as a consequence of calcium imbalance [20]. Recently, a growing number of studies and a recent meta-analysis reported the link between Vit D and sleep [35]. Adequate levels of this hormone seem to be necessary for the maintenance of sleep, reducing the number of nocturnal awakenings [80] while low Vit D levels have been reported to be associated with shorter sleep duration [81,82]. Although the exact mechanism by which Vit D affects sleep regulation is still unclear, the key to this link seems to be the expression of Vit D receptors in the cortical and subcortical areas of the brainstem that are involved in sleep control [83] such as prefrontal cortex [84], cingulate gyrus [85], hippocampus [86], caudate nucleus [87], lateral geniculate nucleus [88], and substantia nigra [83,89].

Interestingly, Vit D is involved in regulating the conversion of tryptophan into 5-HTP and producing melatonin [90] from tryptophan in the brain [67,90]. Melatonin participates in the regulation of circadian rhythms [91] and adjusts the sleep–wake cycle with a consequent positive effect on the quality of sleep [92]. In fact, epidemiology studies found that dietary intake of Vit D was related to the midpoint of sleep, sleep duration, and maintaining sleep [93,94]. In this regard, it seems important to consider that in our sample some patients reported subthreshold depressive symptoms. The data is not surprising because it is well known that anxiety disorders, as well as sleep disturbances, often manifest in comorbidity with depressive symptoms [95]. In fact, other studies indicated that the serotoninergic pathway was implicated in the initiation and maintenance of sleep in different areas of the brain that have been associated with the sleep regulation and that Vit D plays a key function in the regulation of the serotonergic pathway [46] and melatonin production. Moreover, Vit D contributes to neuroplasticity [59] and in the synthesis of other neurotransmitters [96,97,98], confirming the importance of Vit D in sleep but also mood regulation [99].

Most studies evaluating anxiety-related symptoms in different populations indicate an association between low levels of Vit D and anxiety [28,100,101], and some reported that Vit D supplementation is associated with lower anxiety symptoms [102]. In our sample, the regression analysis confirmed the significant association between higher PTH and lower Vit D levels, poor quality of sleep, and anxiety symptomatology emphasizing the close relationship between calcium imbalance and psychopathology in patients with GAD. This finding can be explained by the role of calcium imbalance, especially Vit D, in many brain processes, including neuroimmunomodulation, neuroinflammation, oxidative stress, and neuroplasticity [59] and synthesis of neurotransmitters, all implicated in the pathogenesis of anxiety disorders [96,97,98]. In this regard, Vit D seems implicated in the synthesis of serotonin neurotransmitters through the tryptophan pathways [46]. The alteration of the serotonin synthesis is associated with the prefrontal cortex [103], hippocampal [104] and amygdala dysfunctions [105], brain regions important in regulating network activity, and neural oscillations in anxiety disorders [106,107].

On the other hand, many of the positive effects of Vit D on behaviors might be associated with its ability to regulate both peripheral and CNS immune responses. As noted, anxiety is frequently associated with a low-grade inflammatory status and peripheral increase of inflammatory cytokines [108,109]. As such, Vit D may help reduce anxiety symptoms because of its antioxidant and anti-inflammatory properties. More recently, the preclinical study described the anti-inflammatory and antioxidant effects of the pretreatment with Vit D3 underlying the ability of this vitamin to annul anxiety-like behaviors. Indeed, this effect was accompanied by a decrease in IL-6 levels [110]. Results were replicated in a clinical sample: Vit D supplementation in combination with standard of care improved the severity of anxiety in individuals diagnosed with GAD by increasing serotonin concentrations and decreasing the levels of the inflammatory biomarker neopterin [111].

The results of the present study should be read considering some limitations. First, the cross-sectional study design, the type of patients included (only outpatients), and the relatively small sample size does not allow to generalize to a large proportion of the psychiatric population and preclude establishing causal relationships. In this light, prospective studies are recommended. Second, the self-administered scale and the retrospective nature of the study were affected by the effect of recall bias and represent a structural limitation regarding the assembly and reliability of the data. Third, psychiatric medications are known to trigger symptoms of sleep disorders. Due to heterogeneity in our sample, patients were prescribed different psychotropic medications which would be difficult to control. Hence, it was not possible to examine the association between psychotropic medication and symptoms of sleep disorders. Lastly, the wide overlap of features and neurophysiological systems involved in anxiety and depressive symptoms, even if occurring only in a few patients of our sample, prevented us to examine the unique relationship between calcium imbalance and anxiety disorder. Further studies should assess the role that calcium imbalance plays in this relationship, distinguishing mood disorders from anxiety disorders and using major depressive disorder as a control group.

Despite these limitations, the major strengths of this study are represented by the focus on calcium imbalance and sleep quality in patients with GAD in a real-world setting with broad inclusion criteria. Furthermore, this was the first attempt to evaluate the role and implications of calcium homeostasis in GAD, considering its relationships to sleep and anxiety symptoms. Moreover, the study includes the concomitant assessment of Vit D, PTH, and Ca^++^ levels to assess and analyze the whole metabolism axis. Nevertheless, future large-scale prospective studies are needed to confirm the findings of this study and to better clarify the association between calcium imbalance, sleep quality, and psychopathology severity. Identifying and addressing sleep quality, insomnia, and calcium imbalance may have a positive impact on the prognosis and quality of life of patients with GAD.

## 5. Conclusions

In conclusion, the study found a strong association between levels of parathyroid hormone and Vit D, sleep quality, and anxiety symptomatology in patients suffering from GAD. The study results suggest that patients with GAD and low levels of Vit D and higher levels of PTH exhibit poor quality of sleep and higher levels of anxiety highlighting its impact on the psychopathological burden. Results should suggest that calcium homeostasis may be disrupted in this population but additional prospective studies in real-world settings with direct comparisons between these two conditions are needed. Therefore, it may represent an area of clinical research interest for the future, to reach more patients focused on clinical practice to anticipate a precise diagnosis, manage personalized treatment, and improve prognosis. Indeed, future studies could shed light on the causal and temporal relationship existing between calcium metabolism imbalance, anxiety, and sleep, opening new and interesting frontiers in both clinical and research fields.

## Figures and Tables

**Table 1:** Serum levels cut-off for biological variables.

	Deficiency Level	Intermediate Level	Excess Level
Vit D	<20 ng/mL	21–29 ng/mL (insufficiency level) 30–100 ng/mL (sufficiency level)	>100 ng/mL
Ca^++^	<8.8 mg/dL	8–10 mg/dL	>10 mg/dL
PTH	<15 pg/mL	15–55 pg/mL	>55 pg/mL

Calcium homeostasis imbalance: levels of Vit D < 20 ng/mL, Ca^++^ < 8.8 mg/dL or >10 mg/dL, and PTH <15 pg/mL or >55 pg/mL.

**Table 2:** Socio-demographic and clinical variables.

Total Sample N = 211			
		N (%)	
Sex	Female	108 (51.2)	
	Male	103 (48.8)	
Diploma	yes	161 (76.3)	
Marital status	Single	3 (1.4)	
	Married	96 (45.5)	
	Co-habiting	78 (37.0)	
	Divorced	32 (15.2)	
	Widowed	2 (0.9)	
Occupation	Unemployed	73 (34.6)	
	Employed	133 (63.0)	
	Retired	5 (2.4)	
Family Psychiatric History	yes	136 (64.5)	
		M (SD)	Range
Age		46.91 (13.76)	22–75
Age at onset of GAD		27.82 (11.01)	16–66
HAM-A	Total score	25.6 (13.74)	7–54
PSQI	Quality	1.75 (1.03)	0–3
	Latency	1.52 (1.11)	0–3
	Duration	1.51 (0.94)	0–3
	Efficiency	1.43 (0.99)	0–3
	Disturbances	1.43 (1.04)	0–3
	Medication	1.62 (1.10)	0–3
	Dysfunction	1.64 (1.10)	0–3
	Total score	10.96 (6.18)	0–21
ISI	Total score	14.36 (8.22)	0–33
Calcium level		9.46 (0.38)	8.60–11.00
PTH level		54.64 (20.45)	12.40–87.00
Vit D level		29.42 (25.10)	4.0–332.0

HAM-A: Hamilton Anxiety Rating Scale; ISI: Insomnia Severity Index; M: mean; n: total number; PSQI: Pittsburg Sleep Quality Index questionnaire; PTH: parathyroid hormone; SD: standard deviation; %: percentage.

**Table 3:** Results of Spearman correlation analysis.

	Ca^++^	PTH	Vit D	HAM-A Total	PSQI Quality	PSQI Latency	PSQI Duration	PSQI Efficiency	PSQI Disturbances	PSQI Medication	PSQI Dysfunction	PSQI Total	ISI Total
Ca^++^	-												
PTH	−0.115	-											
Vit D	**0.104**	−0.753 **	**-**										
HAM-A Total	**−0.056**	0.839 **	−0.732 **	**-**									
PSQI Quality	**−0.056**	0.722 **	−0.719 **	0.742 **	**-**								
PSQI Latency	**−0.118**	0.778 **	−0.727 **	0.789 **	0.650 **	**-**							
PSQI Duration	**−0.068**	0.530 **	−0.613 **	0.557 **	0.593 **	0.617 **	**-**						
PSQI Efficiency	**−0.119**	0.663 **	−0.581 **	0.674 **	0.668 **	0.709 **	0.490 **	**-**					
PSQI Disturbances	**−0.112**	0.712**	−0.645 **	0.756 **	0.629 **	0.800 **	0.555 **	0.662 **	**-**				
PSQI Medication	**−0.122**	0.683 **	−0.661 **	0.761 **	0.669 **	0.782 **	0.578 **	0.666 **	0.808 **	**-**			
PSQI Dysfunction	**−0.085**	0.728 **	−0.679 **	0.829 **	0.716 **	0.797 **	0.576 **	0.622 **	0.779 **	0.858 **	**-**		
PSQI Total	**−0.106**	0.784 **	−0.713 **	0.828 **	0.785 **	0.869 **	0.694 **	0.806 **	0.874 **	0.878 **	0.877 **	**-**	
ISI Total	**−0.123**	0.811 **	−0.727 **	0.838 **	0.772 **	0.813 **	0.612 **	0.734 **	0.805 **	0.819 **	0.811 **	0.889 **	**-**

** *p* < 0.01. Significant results are in bold for emphasis. HAM-A: Hamilton Anxiety Rating Scale; ISI: Insomnia Severity Index; PSQI: Pittsburg Sleep Quality Index questionnaire; PTH: parathyroid hormone.

**Table 4:** Linear regression analysis.

Dependent Variable	Independent Variables	Not Standardized Coefficients		Standardized Coefficients		Sign.
		B	Error Standard	Beta	t	
PSQI Total	PTH	0.212	0.015	0.700	140.198	**0.000**
	Vit D	−0.035	0.012	−0.144	−20.909	**0.004**
	Ca^++^	−0.485	0.706	−0.030	−0.686	0.493
^a^ Model 1	Dependent variable: PSQI Total; R^2^ = 0.603; F = 80.752; *p* < 0.001					
HAM-A Total	PTH	0.516	0.030	0.767	170.457	**0.000**
	Vit D	−0.067	0.024	−0.123	−20.783	**0.006**
	Ca^++^	0.813	10.398	0.023	0.581	0.562
^b^ Model 2	HAM-A Total; R^2^ = 0.685; F = 115.137; *p* < 0.001					
ISI Total	PTH	0.306	0.018	0.759	160.781	**0.000**
	Vit D	−0.035	0.015	−0.108	−20.375	**0.018**
	Ca^++^	−10.203	0.862	−0.056	−10.395	0.164
^c^ Model 3	Dependent	variable: ISI Total; R^2^ = 0.672; F = 105.516; *p* < 0.001				

HAM-A: Hamilton Anxiety Rating Scale; ISI: Insomnia Severity Index; PSQI: Pittsburg Sleep Quality Index questionnaire; PTH: parathyroid hormone. Significant results are in bold. PTH and Vit D levels predicted insomnia, higher levels of anxiety, and poor quality of sleep. ^a^ Model 1. Dependent variable: PSQI Total; R^2^ = 0.603; F = 80.752; *p* < 0.001. ^b^ Model 2. Dependent variable: HAM-A Total; R^2^ = 0.685; F = 115.137; *p* < 0.001. ^c^ Model 3. Dependent variable: ISI Total; R^2^ = 0.672; F = 105.516; *p* < 0.001.

## Data Availability

According to European law (GDPR), data containing potentially identifying or sensitive patient information are restricted; our data involving clinical participants are not freely available in a public repository. However, data presented in this study are available on request from the corresponding author.
